# Vocational Value Profiles of Students with Preferential Vocational Interest in Sport and Their Relationship with Personal and Academic Wellbeing

**DOI:** 10.3390/ijerph182211872

**Published:** 2021-11-12

**Authors:** Evelia Franco, Carmen Ocete, Vicente Hernández-Franco

**Affiliations:** Departamento de Educación, Métodos de Investigación y Evaluación, Universidad Pontificia Comillas, 28108 Madrid, Spain; efalvarez@comillas.edu (E.F.); vhernandez@comillas.edu (V.H.-F.)

**Keywords:** vocational guidance, vocational interests, vocational values, sport science, wellbeing

## Abstract

In recent years there has been a significant increase in the number of students who choose to pursue university studies related to the field of sports. However, there are no studies that have investigated the existence of differentiated profiles within students whose preferred vocational area is sport. The main objective of this study was to establish the profiles of students in the second year of Spain Bachillerato whose preferred vocational interest is sport, according to the two representative vocational values: (a) “to have a fun professional activity”; and (b) “to have a professional activity whose schedule makes it possible to reconcile personal and professional life”. In addition, the resulting groups were compared according to their perception of general and academic wellbeing and their identification with the other vocational values. Two hundred and thirty participants (MAge = 17.47; DTAge = 0.669; N = 171; 74.3% male and N = 59; 25.7% female) completed some validated measures. Three clusters emerged which did not differ in terms of general and academic wellbeing. Differences were found though in terms of some vocational values such “to help people”, “to develop one’s entrepreneurial initiative” or “to be self-employed”. The findings invite us to rethink the differences in the specific profiles of vocational values and their impact on employability opportunities, and to consider these approaches in the orientation of students who have among their priority options to study sport sciences.

## 1. Introduction

In recent years, significant changes have taken place in contemporary Western society, reconsidering its career opportunities in a way different than that two decades ago [1]. To this fact must be added the growing evidence on the predictive validity of vocational interests for job performance, leading to a greater consideration of interest assessment in career guidance processes [2]. Following the above and based on the social cognitive theory on which this research is conceptually based [3,4,5,6], vocational interests and vocational values are key dimensions in career development [7]. This is so because in the case of vocational interests, they become predictors of behavior, while vocational values conceptualize preferences, organizing them hierarchically. This implies their consideration in the choice of studies and profession [8,9], as well as being part of vocational guidance programs during the secondary education stage [10,11]. The relevance of understanding personal values when it comes to vocational guidance of high school students seems especially important given the existing association between such values and turnover intentions in the professional setting [12,13]. Furthermore, personal values could also explain relevant human outcomes such as personal wellbeing [14].

Research on the construct of subjective personal wellbeing has experienced a strong increase in recent years [15,16], differentiating between cognitive and emotional components. Although there is no consensus in the literature on their assessment, it seems that the relationships they maintain with other psychological variables describe differently [17]. From the cognitive point of view, life satisfaction is an evaluation that reflects a long-term perspective and is consciously made by the person about the circumstances of his or her life; therefore, it may reflect conscious values and goals [18]. Furthermore, the literature states that there must be some degree of convergence between life satisfaction and emotional wellbeing because both depend on subjective appraisal. Taking these aspects into account, judgments about satisfaction depend on the comparisons that the subject makes between his or her life circumstances and a standard that he or she considers adequate. This last nuance is important since it is not an externally imposed standard, but a self-imposed criterion [19], and personal wellbeing appears when the person considers that he or she lives in congruence with his or her beliefs and values [20]. The relationships of these constructs have been studied from different contextual perspectives. Specifically, the university context is one of them, since it has been determined as fundamental that higher education ensures the academic wellbeing of its students [5,20], so that the transition from secondary to university education is a process of comprehensive training [21]. Despite the numerous investigations contextualized in university education on vocational interests, few have focused on these issues related to systematic learning processes (social relations, arts, philosophy, etc.) necessary to achieve better human development [22].

Focusing on studies related to physical activity and sport (including the degrees of Specialist Teacher in Physical Education (PE), former Bachelor of Physical Activity and Sport Sciences and current Physical Activity and Sport Sciences Degree) and their unquestionable rise in relation to the number of students enrolled [23], it is worth asking about the vocational dimension of future professionals and whether there is a vocational component that predisposes them to it [24]. In the study carried out by Hernández-Franco and Franco [24], it was shown that students with a marked vocational interest in this field have a high interest in the health area. In turn, they have a predilection for activities involving the performance of physical tasks and higher scores than other young people in other areas of vocational interests such as protection and security and a low interest in languages. In the study developed by Pérez, Requena and Zubiaur [25], students with a marked vocational interest in physical activity and sport showed a high intrinsic interest in sport and a higher level of physical activity than other young people. Despite these approaches to the vocational identity of this population, there are no studies that have investigated “ad intra” the existence of differentiated profiles within students whose preferred vocational area is sport.

As mentioned above, one of the resources used for guidance processes to obtain empirical indicators of vocational identities are vocational guidance programs in the secondary and baccalaureate stages of education. An example of this type of initiative is the “Orion Project” of the Universidad Pontificia Comillas de Madrid (www.upcomillas.es/myvip/alumnos/, accessed on 2 November 2021). Its main objective is to offer updated information to students and to the network of guidance counselors at participating schools, helping students in the third and fourth years of ESO and first and second years of Bachillerato to make decisions about their vocational options [26]. The importance of this scenario lies in the fact that the different vocational groups into which the set of professions present in our society can be classified are defined by specific vocational interest profiles and values.

The scarce scientific literature on the variables that young people take into consideration when choosing their university studies at the end of secondary education, or if their vocational profile differs from the profile of other students [26] leads us to search for scientific evidence on which to base future practical applications. Considering the association between personal values and turnover intentions [12], gaining some knowledge about how these hypothetical profiles behave in terms of other vocational values as well as in their wellbeing seems crucial to better understand which professional options might be more suitable for each group and, in turn, to decrease dropout rates among physical activity and sport professionals. The present work pursues thus to answer the questions: (a) are there different profiles in terms of representative vocational values among high school students whose main vocational interest is sport; and (b) do these profiles differ in terms of other vocational values and wellbeing experience? 

For this reason, the objectives of this study are, firstly, to establish the profiles of second-year high school students whose preferred vocational interest is sport, according to their scores on the two vocational values which are representative of this group of young people: (a) “to have a fun professional activity”; and (b) “to have a professional activity whose schedule makes it possible to reconcile personal and professional life”. In addition, the main substantive contribution of this work is to compare the resulting groups according to their subjective personal and academic wellbeing, and their identification with the rest of their vocational values. Given the different nature of the two representative values for this group of students, and believing that they can be differently combined, it is hypothesized that different profiles might emerge according to the two representative vocational values of this group of students. Due to the lack of previous studies addressing this research question, we cannot hypothesize either which profiles will emerge or how they will differ in terms of the rest of vocational values and wellbeing. This is actually one important and innovative contribution of the present work. 

## 2. Materials and Methods

### 2.1. Design and Participants

The present study is based on a quantitative methodological approach, with a non-experimental, exploratory and cross-sectional descriptive research design, using the survey technique. Non-probabilistic convenience sampling was carried out considering existing research about sample size requirements for cluster analysis, factor analysis, and MANOVA [27]. From a total of 4715 high school students from the Community of Madrid (44% male and 56% female) (N_state_ = 1124, N_private_ = 3591; aged between 17 and 20 years (M = 17.37; SD = 0.65)), one subsample of 230 students (4.8%, M = 17.47; SD = 0.669; N = 171; 74.3% males and N = 59; 25.7% females) indicated that studying Physical Activity and Sport Sciences was among their priority options and thus formed the final sample of this study.

### 2.2. Instruments

Sociodemographic variables. Students indicated gender and age. 

Vocational values. To identify the vocational values of the participants, the Vocational Values Questionnaire (CERVO_2010 [28,29,30]) was used. This is a questionnaire of expressed values consisting of fourteen items that are answered on a Likert-type scale from 0 to 10 according to their degree of preference for each of them, with 0 corresponding to “rejection” and 10 to “it is one of my favorites”. They come pre-given by the heading: “When I think of my ideal future job, I mainly hope to get…”. In previous work, the internal consistency of the scale (Cronbach’s alpha) was 0.718 [29].

**Figure 1:**
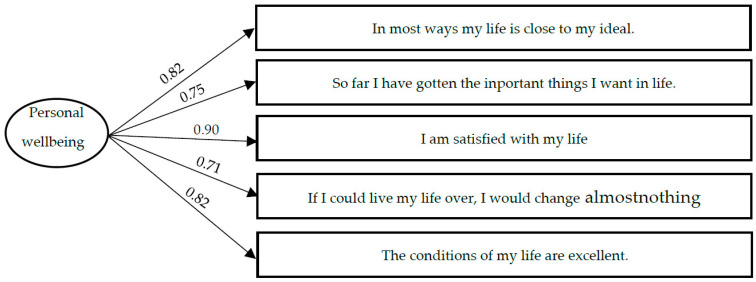
Measurement model of the Subjective Personal Wellbeing scale.

Subjective personal wellbeing. To analyze the personal wellbeing of the participants, we used the Spanish version of the Satisfaction with Life Scale (SWLS; [31]), adapted to Spanish and validated in an adolescent population [17]. This scale consists of five items that assess the overall judgment that people make about their life satisfaction (e.g., “in most aspects, my life is as I want it to be”). Although the original version of the scale offered seven response options on a Likert scale, in the present study it was decided to expand the response options to eleven on a scale of 0 to 10. This decision was based on the fact that our study sample was administered a battery of tests, including this scale, in order to investigate the wellbeing and vocational values of adolescents. Due to the diversity of response scales of the tests used, it was decided to homogenize the number of response values in some instruments in order to minimize the possible confusion generated by the different response scales. Thus, in the version used in the present study, the responses range from 0 to 10, where 0 = “strongly disagree”, and 10 = “strongly agree”. Although this scale had already been validated, due to the change performed in the responses scale, its psychometric properties were tested again by means of a confirmatory factor analysis in which a unifactorial structure with five indicators was tested. The resulting model showed adequate fit indices (χ^2^/gl = 32.98; CFI = 0.98; TLI = 0.97; GFI = 0.98; SRMR = 0.06). Regression weights of the indicators on the latent variant of between 0.75 and 0.90 were obtained. Figure 1 shows the items that made up the scale and the regression weights of each item on the latent variable. Cronbach alfa was 0.89. The Spanish version of this instrument can be found in the Table A1.

**Figure 2:**
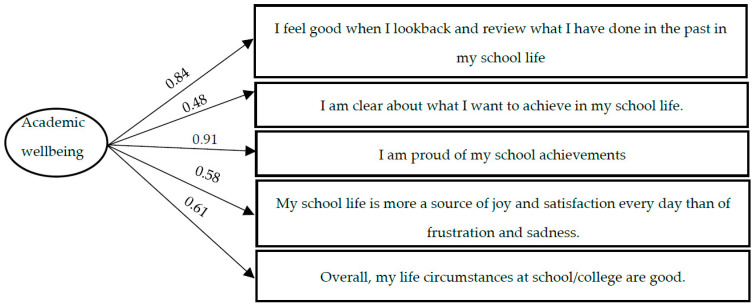
Measurement model of the Subjective Academic Wellbeing scale.

Subjective academic wellbeing. To analyze the academic wellbeing of the participants, the Satisfaction with Life Scale (SWLS; [31]) was adapted to the academic context. This adaptation consisted of incorporating slight linguistic modifications to the original items of the scale to reflect the academic context. The scale consists of five items that assess the students’ overall judgment of their satisfaction with their academic life (e.g., “on the whole, the circumstances of my life at school/institute are good”). Its psychometric properties were tested by means of a confirmatory factor analysis in which a unifactorial structure with five indicators was tested. The resulting model showed adequate fit indices (χ^2^/gl = 16.11; CFI = 0.90; TLI = 0.90; GFI = 0.98; SRMR = 0.05). Regression weights of the indicators on the latent variant of between 0.46 and 0.93 were obtained. When looking at the suggested modification indices, a high correlation between the errors of two of the indicators was apparent. After verifying the similarity between both items (“my school life is every day more a source of satisfaction and joy than of frustration and sadness” and “on the whole, the circumstances of my life at school/institute are good”), it was decided to correlate both errors. Although the initial model was adequate, this new model showed better fit indices (χ^2^/gl = 16.11; CFI = 0.97; TLI = 0.97; GFI = 0.98; SRMR = 0.05). Figure 2 shows the items that made up the scale and the regression weights of each item on the latent variable. Cronbach alfa was 0.80. The Spanish version of this instrument can be found in the Table A2.

### 2.3. Procedure

The collaboration of the participating centers was requested based on convenience sampling within the network of centers that have participated in the different editions of the Orion Project, and a letter was sent to each center briefly explaining the objective of the study and requesting the collaboration of their students. After obtaining the pertinent permissions and consents in accordance with the ethical guidelines of the American Psychological Association [32] and presenting the questionnaire to the students, the participants completed the on-line questionnaire using the form designed for this purpose by the Orion Project of the Comillas University from Madrid (Spain). It is very important to emphasize when considering the reliability and validity of the data obtained in this work that the responding students have participated voluntarily in this study and without any repercussion on the grade of their subjects. The questionnaires were completed during school hours in on-line format in the center’s own computer classrooms, supervised by their counselors, within the framework of the Academic and Professional Guidance Plan, with an approximate application time of 50 min. They have been presented to them as a meaningful activity of vocational reflection and as an instrument of help and clarification in the process of planning their professional career, offering them with due confidentiality the opportunity to individually discuss the results with their course tutors and with the educational counselor of their center. This work followed the guidelines of the ethical principles of the Declaration of Helsinki. The center’s counselor, a member of the research team, collected the informed participation consents and was the person who informed the participating students and their families about the procedure and data treatment.

### 2.4. Data Analysis

First of all, normality distribution of the data was assessed using the Shapiro–Wilk statistic, with normality assumed when *p* > 0.05. Then, a cluster analysis was performed following the two-stage procedure designed by Hair et al. [33]. This procedure consists of firstly performing an exploratory analysis through ahierarchical cluster analysis using the Ward method. In the second phase, the k-means test is used to contrast the solution both in the subsample used in the test performed with the Ward method and in the other subsample that had not been used. Once the profiles had been identified, in order to check whether there were significant differences in these profiles in terms of their vocational values, a multivariate analysis of variance (MANOVA, Wilks’ Lambda test) was carried out, and then the corresponding univariate tests were performed. Finally, Scheffé’s test was used for post hoc comparisons, and the effect size of the differences was calculated. The SPSS 24.0 statistical package was used.

## 3. Results

### 3.1. Profiles Establishement

After conducting the hierarchical cluster analysis, agglomeration coefficients suggested that a three-cluster solution was suitable given the incremental increases observed in the aforementioned coefficients from five to four clusters and from four to three (when three clusters were merged to two, however, there was a sharp increase in the aggloremation coefficients). The dendogram also supported a three-cluster solution for classifying participants according to the two chosen vocational values (“to have a fun professional activity”; and “to have a professional activity whose schedule makes it possible to reconcile personal and professional life”). 

**Figure 3:**
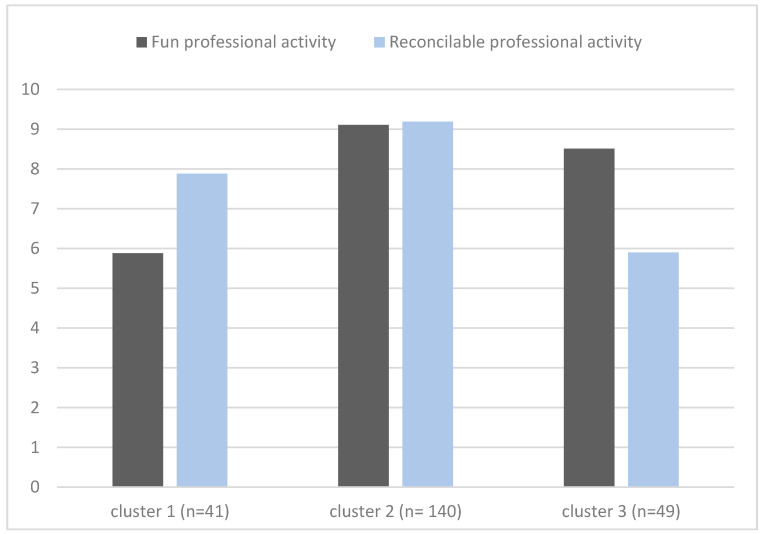
Mean clusters scores.

Based on the scores obtained in the two variables to form the clusters, the following labels were assigned to the three groups: (a) cluster group 1 (*n* = 41; 73.2% boys); (b) cluster group 2 (*n* = 140, 71.4% boys) and (c) cluster group 3 (*n* = 49; 83.7% boys). Figure 3 represents the graphical results of the solutions of the three clusters as a function of the mean scores on these two vocational values. No differences were found in the distribution between men and women among the three resulting clusters (Chi-square = 2.890; *p* = 0.236). Multivariate differences were found between the mean vocational value profiles of the three clusters (Wilks’ Lambda = 0.174; F(4, 452) = 157.644; *p* < 0.001; η^2^*p* = 0.582). The mean vocational value profile scores for each cluster as well as the univariate differences in the between-cluster contrasts are presented in Table 1. Cluster 2 (M = 9.11; SD = 0.93) showed higher values on the vocational value “having a fun professional activity” than cluster 3 (M = 8.51; SD = 1.18), which, in turn, showed higher scores than cluster 1 (M = 5.88; SD = 1.26) on this same value. As for the value “having a professional activity that is reconcilable with personal life”, cluster 2 (M = 9.19; SD = 0.82) also showed the highest scores, followed by cluster 1 (M = 7.88; SD = 1.36) and cluster 3 (M = 5.90; SD = 1.30) in this order. These differences allowed us to conjecture the labeling of the clusters that we have collected in Table 2 and that we will discuss in more detail in the discussion.

### 3.2. Differences in Personal and Academic Wellbeing

Regarding wellbeing, multivariate analysis reflected the absence of significant differences between clusters (F(4444) = 0.55, *p* = 0.70). Subsequent univariate analyses confirmed that there were no differences in either personal wellbeing (F(2) = 0.86, *p* = 0.42) or academic wellbeing (F(2) = 0.12, *p* = 0.89).

### 3.3. Differences in Vocational Values

The MANOVA performed with the clusters as the independent variable and the rest of the vocational values that had not been used in the conformation of these clusters as the dependent variables also revealed significant differences between the groups (Wilks’ Lambda = 0.159; F(28, 428) = 23.060; *p* ≤ 0.001; η^2^*p* = 0.601). As can be seen in Table 1, participants in cluster 2 showed higher scores on a set of vocational values relative to cluster 1: “helping people”, “developing my entrepreneurial initiative”, “having the conviction to do something useful and important”, “dynamism and varied activity”, “self-employment”, “cultivating my personal potential”. In addition, cluster 3 showed greater identification than cluster 1 in the values: “having the conviction to do something useful and important” and “dynamism and varied activity”. 

## 4. Discussion

Considering the association between personal values and turnover intentions [12], it seems crucial to better understand which professional options might be more suitable for each group. Given the different nature of the two representative values for this group of students, it is hypothesized that different profiles might emerge according to the two representative vocational values. The aim of this study was to establish the vocational profiles of the students of the second year of the Spanish Bachillerato who have as their preferred interest the vocational area of sport from the perspective of their two preferred vocational values: (a) to enjoy a fun professional activity, which allows me to experience new sensations, tackling different challenges and risks, whether physical, economic, etc., and which allows me to feel enthusiastic all the time about what I do without ever getting bored, and (b) to have a good schedule, which allows me to reconcile my personal and professional life, having a flexible schedule and enough free time to devote to my hobbies and social activities: family, friends, volunteering, travel, sports, music, reading, cinema, theater, new studies, etc. 

Table 2 displays the three profiles established in the cluster analysis. 

As shown in Table 2, three profiles emerged. The first of these showed low scores on the value “to have a fun professional activity” and high scores in “to have a professional activity whose schedule makes it possible to reconcile personal and professional life” and was labelled as “teaching”. The second profile showed high scores in “to have a fun professional activity” as well as in “to have a professional activity whose schedule makes it possible to reconcile personal and professional life”. This profile was labelled as “multiprofessional” Lastly, the third profile, labelled as “Sport management”, was characterized by a relatively high score on the value of having a fun professional activity, and a relatively low score on the value of this activity being reconcilable with personal life.

“Teaching” was the first profile identified through the analysis. It is worth mentioning that being a PE teacher has traditionally been the main occupation of university graduates in this area according to Hardman [34] and Campos [35]; however, studies such as the one carried out by Gómez-Tafalla et al. [36] show how there is a progressive diversification in the professional opportunities of CCAFYD graduates, with a decrease in professionals who occupy teaching-related positions and an increase in those who work in the management and direction of sports facilities.

This professional career path towards teaching could be aligned with the preference for having a work activity that allows the reconciliation of personal life and work, especially if it is carried out in public educational centers. We agree with Sánchez and Rebollo [37], therefore, that the factor of job stability has conditioned and continues to condition in a decisive way the choice of professional field, considering teaching and public management as areas of work with greater stability, while private management, high performance and the field of health as areas of lesser stability. On the other hand, as pointed out by Campos et al. [38], it is quite common for recent graduates to enter the labor market as sports technicians in schools or sports clubs. These contexts, designed to occupy the leisure time of children and adolescents, could well respond to the importance of young people who pursue sports-related studies to perform a fun professional activity. It is relevant to mention that many professionals in this field combine for years their work as PE teachers with that of monitors and technicians. 

Finally, the first profile that we have conceptualized as “Teaching” was characterized in comparison with the other two profiles by relatively high scores in terms of their professional activity being reconcilable with their personal life and relatively low scores in the importance of the professional activity being fun. This group of young people also showed the lowest scores for developing their entrepreneurial initiative or having a dynamic and varied work activity. These results coincide with those presented by Castañeda-Vázquez et al. [39], which show numerous motives that encourage interest in teaching, ranging from altruistic motivations related to intrinsic motivation and teaching vocation, to more pragmatic motives centered on the benefits of the teaching profession, such as the stability and security offered by the civil service, salary or vacations. Supporting these results, the research work of Latorre and Blanco [40] showed interest mainly in more extrinsic motivations, such as the absence of other professional opportunities or the material conditions of the profession. As previously described, the second profile showed high scores in “to have a fun professional activity” as well as in “to have a professional activity whose schedule makes it possible to reconcile personal and professional life”. This profile was labelled as “multiprofessional”. We could think that this professional profile corresponds to people taking part in the different occupations that characterize the area of sport. While working as a PE teacher as the main occupation would guarantee them an activity that could be reconciled with their personal life, working as a monitor as a secondary professional activity, would provide their professional life with the fun character that they also value.

The analysis also allowed us to establish a third profile that we have conceptualized as “Sport management”, characterized by a relatively high score on the value of having a fun professional activity, and a relatively low score on the value of this activity being reconcilable with personal life. This profile stands out in the values of “entrepreneurial initiative” and “dynamism and varied activity”. These results coincide with the work of Gómez-Tafalla et al. and Sánchez and Rebollo [36,37], where a profile linked to satisfying the experienced social demand, in which the practice of physical-sporting activity evolves in areas other than education, such as the field of sports management, is presented. According to Gambau [41], this represents 27.93% of the identified national outlets, corresponding to the first most identified professional outlet of the degree in sport sciences, followed by sport performance (24.32%) and teaching of PE and sport (18.47%).

This requires more specialized and effectively trained professionals for the organization of events and competitions, management of human talent and creation of projects, among other functions, performed in private entities such as sports service companies, associations, clubs and/or sports federations. Furthermore, according to Gómez-Tafalla [42] this profile is related to the appearance of the “economic retribution” factor, since there is the possibility of obtaining an important economic income, assuming, in turn, the corresponding uncertainty of a sports company or the sports results of a team or athlete. In this sense, we agree with Salgado-Barandela [43] on the need to influence the profile and delimitation of the functions of sports managers with the aim of differentiating competencies among professionals with university training. 

The findings on the absence of significant differences in subjective personal wellbeing suggest a weak association between the vocational values of having a fun professional activity and having an activity that is compatible with one’s personal life and wellbeing. Previous studies like that of González-Serrano [44] have shown how the context of sport practice can influence subjective personal wellbeing. According to the results of the present study, it seems that the academic and personal wellbeing of students with a preference for sport studies is not conditioned by their vocational preferences. However, according to the literature, there is evidence that professional experience in adult life may be related to the quality of life associated with work, as Salazar-Estrada point out [45]. Similarly, there are numerous studies proposing that personal wellbeing or distress may be affected by work experience, as we can appreciate in Franco et al. and in Salazar-Estrada [45,46]. The findings of this study, together with those suggested in the aforementioned works, suggest the need to explore at what point this association between perceptions of the personal and professional spheres occurs. Although a person’s vocational values do not seem to define the tendency to experience personal wellbeing, it would be interesting to know which factors linked to the work environment affect wellbeing in physical activity and sport science professionals. 

This is consistent with the evidence on how the context of sport practice affects subjective personal wellbeing as discussed by Guerrero-Barona et al. [47] and may favor the way in which the person self-regulates emotionally. It seems to be interesting to identify which elements are determinants of academic wellbeing, based on the results obtained regarding the relationship between life satisfaction and an active lifestyle, where psychological wellbeing in general is related to the practice of physical-sporting activity as pointed out Torregrosa et al. [48].

It is necessary to highlight the relevance of continuing to deepen these findings, since several investigations such as that of Vera et al. [49] support that feeling satisfied with oneself can impact the performance of students or the way they face demanding situations, such as in this case, the choice of university studies. Likewise, these data constitute a challenge for educational institutions, since according to Barrantes-Brais and Ureña-Bonilla and García-Viniegras [50,51], incorporation into university life can sometimes be a challenge to students’ abilities and skills. Obtaining data on their degree of wellbeing could become a protective factor by positively influencing their quality of life. 

This study presents some limitations that should be mentioned. Firstly, despite the large size of the sample which was initially approached, the final subsample participating in this study was much more limited by focusing only on students with a vocational interest in the area of sports. Given the difficulty of recruiting participants interested in sport in ordinal high schools (less than 5% of the students approached reported that their main interest was sport), it would be advisable for future studies in this line to contact university students from the Physical Activity and Sport Sciences Degree. Secondly, it is widely acknowledged that family plays a significant role in the students’ decision for a professional career. However, in this study, family influence was not deemed as a confounding factor. Considering the close association between the choice of a professional career and vocational values, it would be interesting for future studies to address how family-related outcomes might affect vocational values. Lastly, it should be mentioned that the analyses were performed on the basis that ordinal variables can be treated as numerical variables. While there are some arguments in favor of exclusively considering these variables as categorical, there is also a strong line supporting dealing with ordinal variables as continuous, according to Robitz [52].

Despite these limitations, this study makes a relevant contribution to the existing literature. Its main strength is the use of a methodological approach (cluster analysis) that is not widely used in this field and can shed some interesting light on the field. This methodology allows an analysis more centered on the person than on the variables, since the clusters are actually established according to participants’ characteristics. The advantage of this method lies in the possibility of identifying naturally occurring combinations of students’ vocational values. Furthermore, it means a first approach to the analysis of the association between vocational values and wellbeing. 

With a view to future lines of study, considering that subjective wellbeing encompasses the evaluation of the individual’s resources, among which is his or her ability to control and manage emotions, it would be interesting to approach the formation of beliefs from the perspective of the Theory of Planned Behavior postulated by Azjen [53], applying this topic of study in this context of secondary education. It would also be of great interest to contrast the results established here by means of longitudinal studies, with a quantitative and qualitative design. This type of work will allow us to analyze the evolution and characterization of the vocational values of students belonging to the same cohort of second-year high school graduates who choose to study for a degree in CCAFYD during the four years of their undergraduate studies. It must be considered that current education and the continuous social changes produced require increasingly specific professional profiles that meet the needs of the current population, as pointed out by Ocete et al. [54]. For example, the inclusion of people with disabilities in sports, either in the context of leisure and recreation or in education, forces graduates in sport science to acquire not only specific skills but also to show values that predispose towards this type of activities, not always accepted and assumed by teachers or sports technicians. Knowing them in advance could facilitate specific guidance to work in contexts of application of adapted sports or processes of sports inclusion of people with disabilities. Furthermore, we believe that it would be useful to develop specific instruments in our country to evaluate the verbal interests shown by graduates in Physical Activity and Sport Sciences towards the main occupational activities to which the different postgraduate courses linked to these studies give access. This analysis would help provide students with better professional orientation during their university studies. Lastly, it would be interesting to explore how these profiles evolve. Carrying out longitudinal studies to analyze the stability of the vocational profiles after university and once participants are developing their professional careers would help understand to what extent these profiles can actually be linked to the different professional fields.

## 5. Conclusions

In conclusion, the findings shown in this work have allowed us to identify and reflect on the “ad intra” profiles that we can find among the students in the second year of the Spanish Bachillerato interested in the vocational area of sport from the perspective of their vocational value profiles, and show the close relationship that these profiles have with the professional activities that they will be able to perform in the future. From all that has been exposed in our work, we want to emphasize the importance of accompanying students in these courses in the reflection on their professional interests as a step prior to the choice of studies or profession, and we trust that the findings that this work reveals can be taken into consideration in the vocational guidance of Secondary and Baccalaureate students interested in the studies of the vocational area of sport and contribute to facilitate a better transition to these higher studies.

## Figures and Tables

**Table 1:** Differences in vocational values as a function of membership clusters.

Vocational Values	Cluster 1 (*n* = 41) M (SD)	Cluster 2 (*n* =140) M (SD)	Cluster 3 (*n* = 49) M (SD)	*F*	*p*	Partial η^2^
To have a fun professional activity	5.88 (1.26)_1,2,3_	9.11 (0.93)_1,3_	8.51 (1.18)_1,2_	170.97	0.001	0.601
To have a professional activity whose schedule makes it possible to reconcile personal and professional life	7.88 (1.36)_2,3_	9.19 (0.82)_1,3_	5.90 (1.30)_1,2_	159.47	0.001	0.584
Personal wellbeing	7.04 (1.64)	7.12 (1.58)	7.20 (1.47)	0.12	0.888	0.002
Academic wellbeing	6.44 (1.52)	6.81 (1.73)	6.66 (1.38)	0.86	0.424	0.005
To reach fame and social prestige	5.12 (2.28)	5.91 (2.59)	5.83 (2.75)	1.76	0.173	0.015
To get wealth and economic benefit	6.76 (2.05)	7.01(2.14)	6.80 (2.08)	0.32	0.721	0.003
To help people	6.78 (1.82)_2_	7.89 (1.70)_1_	7.24 (1.90)	7.92	0.001	0.065
To develop one’s entrepreneurial initiative	5.33 (2.05)_2_	6.91 (1.91)_1_	6.34 (2.17)	11.49	0.001	0.092
To become a civil servant or get a permanent job as an employee	5.41 (2.24)	6.11 (2.38)	5.34 (2.70)	2.55	0.080	0.022
To have the conviction of doing something useful and important	6.98 (2.78)_2,3_	7.56 (2.01)_1_	7.24 (1.65)_1_	11.14	0.001	0.089
To enjoy dynamism and varied activity in one’s work tasks	6.24 (1.95)_2,3_	8.00 (1.84)_1_	7.71 (1.83)_1_	16.15	0.001	0.125
To perform managerial functions	5.02 (2.25)	5.89 (2.50)	6.10 (2.41)	2.84	0.060	0.024
To be self-employed	4.76 (2.32)_2_	5.71 (2.23)_1_	5.05 (2.04)	3.97	0.020	0.034
To develop transcendent values	4.86 (2.43)	5.76 (2.73)	6.20 (2.26)	3.31	0.038	0.028
To cultivate one’s personal potential	6.53 (1.58)_2_	7.54 (2.07)_1_	7.46 (1.84)	5.08	0.007	0.043
To achieve professional excellence	7.31 (1.62)	8.05 (1.86)	7.54 (2.15)	3.36	0.036	0.029

Note: The subscripts indicate the number of clusters with which significant differences were found in the post hoc tests (Sheffe) at the 5% level. The absence of a subscript indicates that no significant differences were found with any of the other two clusters.

**Table 2:** Conceptualization of the groups according to the resulting combination of their two preferred vocational values.

Vocational Values		Low	High
TO HAVE A PROFESSIONAL ACTIVITY WHOSE SCHEDULE MAKES IT POSSIBLE TO RECONCILE PERSONAL AND PROFESSIONAL LIFE	Low		Profile 3 Sport management N = 41
	High	Profile 1 Teaching N = 49	Profile 2 Multi-professional N = 140

## Appendix A

**Table A1:** Spanish and English versions of the Subjective Personal Wellbeing Scale.

Bienestar Personal	Personal Wellbeing
1. En la mayoría de los aspectos de mi vida personal es como quiero que sea	1. In most aspects of my personal life it is how I want it to be
2. Hasta ahora he obtenido las cosas importantes que quiero en la vida	2. So far I have obtained the important things I want in life
3. Estoy satisfecho con mi vida	3. I am satisfied with my life
4. Si pudiera vivir mi vida de nuevo, me gustaría que todo volviese a ser igual	4. If I could live my life again, I would like everything to be the same again
5. En conjunto las circunstancias de mi vida personal son buenas	5. Overall, the circumstances of my personal life are good

**Table A2:** Spanish and English versions of the Subjective Academic Wellbeing Scale.

Bienestar Académico	Academic Wellbeing
1. Me siento bien cuando vuelvo la vista atrás y reviso lo que he hecho en el pasado en mi vida escolar	1. I feel good when I look back and review what I have done in the past in my school life
2. Tengo claro lo que quiero conseguir en mi vida escolar	2. I am clear about what I want to achieve in my school life
3. Me siento orgulloso de mis logros escolares	3. I am proud of my school achievements
4. Mi vida escolar es cada día más una fuente de alegría y satisfacción que de frustración o tristeza	4. My school life every day is more a source of joy and satisfaction than of frustration or sadness
5. En conjunto, las circunstancias de mi vida en el colegio/instituto son buenas	5. Overall, my life circumstances at school/institute are good
